# Therapy of infected wounds: overcoming clinical challenges by advanced drug delivery systems

**DOI:** 10.1007/s13346-021-00932-7

**Published:** 2021-02-20

**Authors:** Pia Kaiser, Jana Wächter, Maike Windbergs

**Affiliations:** Institute of Pharmaceutical Technology and Buchmann Institute for Molecular Life Sciences, Goethe University Frankfurt, Max-von-Laue-Str. 15, 60438 Frankfurt am Main, Germany

**Keywords:** Wound infection, Bacterial biofilm, Drug delivery systems, Antimicrobial resistance, In vitro wound models, Wound dressings

## Abstract

In recent years, the incidence of infected wounds is steadily increasing, and so is the clinical as well as economic interest in effective therapies. These combine reduction of pathogen load in the wound with general wound management to facilitate the healing process. The success of current therapies is challenged by harsh conditions in the wound microenvironment, chronicity, and biofilm formation, thus impeding adequate concentrations of active antimicrobials at the site of infection. Inadequate dosing accuracy of systemically and topically applied antibiotics is prone to promote development of antibiotic resistance, while in the case of antiseptics, cytotoxicity is a major problem. Advanced drug delivery systems have the potential to enable the tailor-made application of antimicrobials to the side of action, resulting in an effective treatment with negligible side effects. This review provides a comprehensive overview of the current state of treatment options for the therapy of infected wounds. In this context, a special focus is set on delivery systems for antimicrobials ranging from semi-solid and liquid formulations over wound dressings to more advanced carriers such as nano-sized particulate systems, vesicular systems, electrospun fibers, and microneedles, which are discussed regarding their potential for effective therapy of wound infections. Further, established and novel models and analytical techniques for preclinical testing are introduced and a future perspective is provided.

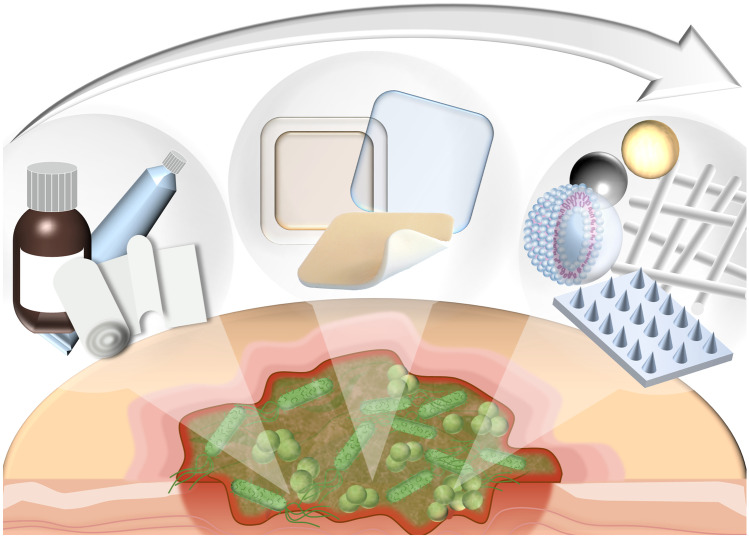

## Introduction

**Fig. 1:**
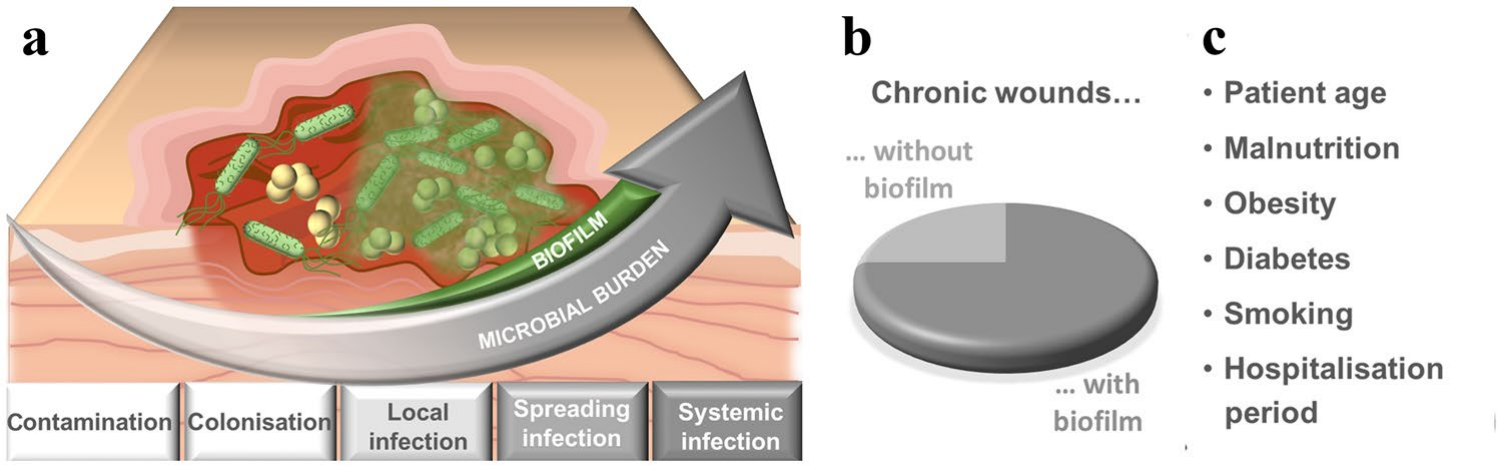
**a** Schematic illustration of the wound infection continuum consisting of 5 stages, from the stage of local infection bacteria organize themselves in biofilms. **b** Biofilms show a prevalence of 75% in chronic wounds. **c** Risk factors promoting wound infection

In recent years, the incidence of chronic wounds and severe wound infections has steadily increased. While normal acute wounds heal without therapeutic intervention, chronic wounds are characterized by necrotic tissue, an increased pH value, and a high concentration of metalloproteases, impeding the course of physiological healing cascades [1, 2]. Such wounds provide a favorable environment for invasion and proliferation of pathogens, and therefore, wound infections often occur. The prevalence for all chronic wounds was assumed to be 1 to 2% of the population in 2018, with healthcare spending up to $96.8 billion in the USA [3]. Apart from the economic impact, chronic wounds inflict a significant decrease in the patients’ quality of life. One cause for the impaired healing of infected wounds is biofilm formation, a local manifestation of wound infection [4], with an overall prevalence of 75% (Fig. 1) [5]. Risk factors, such as patient age, malnutrition, obesity, diabetes, as well as smoking promote wound infection (Fig. 1) [6, 7]. Due to the demographic change towards an elderly society with patients with multi-morbidities, there is an increasing health-economic burden and a growing humanitarian interest in effective treatment of wound infections.

While microbes can be found in every open wound [8, 9], their presence does not necessarily manifest in a wound infection. A wound infection provokes an immunological host response and is characterized by e.g., local inflammation, swelling, erythema, or pain. The wound microbiome consists of bacterial pathogens as well as of fungi, which either interact with the bacteria and may increase antibiotic resistance or which are even primary pathogens themselves. The most frequently found bacterial strains in infected wounds are *Staphylococcus aureus* and *Pseudomonas aeruginosa* [10–12], while the most common fungi genus is *Candida *spp. [13]. The course of infection in a wound can range from colonization of the wound without affecting healing, to a systemic infection with sepsis and organ dysfunction at worst [8, 14]. This progression, characterized as the wound infection continuum, is divided into contamination and colonization as well as local and spreading infection, eventually culminating in systemic infection (Fig. 1). Local, spreading and systemic infection require either topical and/or systemic therapeutic intervention [8, 9, 15]. According to the wound infection continuum defined by the *International Wound Infection Institute*, bacteria organize themselves in biofilms initiated in the stage of local infection [15]. In contrast to free-floating (planktonic) bacterial cells, biofilms are structured, in nature often polymicrobial populations that usually adhere to the wound and are surrounded by extracellular polymeric substances (EPS) [16].

The presence of biofilms poses a particular challenge in the treatment of infected wounds [17]. On the one hand, biofilm bacteria are less susceptible to the human immune defense system due to the mechanical EPS barrier and antiphagocytic properties of the biofilm matrix. On the other hand, biofilms exhibit several mechanisms to develop antibiotic resistance. Firstly, the EPS acts as a mechanical barrier to the permeation and action of antimicrobial agents. Secondly, the biofilm environment enables bacterial communication and thus promotes phenotype changes [8, 18]. A tight growth pattern and slow metabolic rates lead to the formation of a heterogeneous population of cells in different growth states, additionally contributing to the increased tolerance against antimicrobial therapy [17]. As a result, biofilms can persist over a long period of time and boost chronification of a wound, as well as cause other local or remote invasive infections by dispersing biofilm fragments, planktonic bacteria, and microcolonies of mature biofilms [8, 14, 18]. Bacteria within biofilms are up to 1000 times more resistant to conventional antimicrobial agents than those in a planktonic state [8, 9, 18]. Due to these resistances and tolerances towards antibiotics and biocides, biofilms impede appropriate treatment and, consequently, prolong the wound healing period [19].

## Therapy of infected wounds

Providing and maintaining a clean and moist wound environment supporting the physiological wound healing process as well as preventing wound infections is considered the primary goal in general wound management. In case of wound infections, first priority is to achieve a reduction in microorganism quantity or virulence in the wound by debridement, therapeutic cleansing, and administration of antimicrobials.

### Debridement

The presence of necrotic tissue and foreign material in wounds provides a medium for infection; hence, its removal through debridement is a major and widely used method to prevent or reduce bacterial growth. The remaining viable tissue is thereby enabled to undergo the normal wound healing phases [20]. Next to the surgical and the conservative sharp debridement, a variety of alternative techniques exists nowadays including mechanical, autolytic, enzymatic, chemical, and biosurgical wound debridement [15, 21].

Regardless of the type of debridement, it is extremely difficult to remove the entire bacterial bioburden, especially in case of biofilms. This is due to the fact that biofilms are strongly adherent to the surrounding tissue and that its formation is not only limited to the wound surface but also appears in deeper layers of the wound bed [22]. Additionally, depending on the present pathogens, mature biofilms can regrow within just 72 h after debridement, and thus, repetitive debridement is recommended [23]. Even so, debridement alone cannot remove all microorganisms from the wound [24], but the resulting biofilm disruption is associated with a higher susceptibility to outer factors, such as antiseptics or antibiotics [25]. Consequently, debriding the wound in combination with therapeutic cleansing and the application of topical antimicrobial therapeutics is reasonable.

### Therapeutic cleansing

Therapeutic wound cleansing aims to remove problematic excessive or obviously infected exudate, contaminations by foreign body, dirt or bacteria, as well as slough or necrotic tissue [26]. According to Leaper et al., the application of therapeutic wound cleansing solutions even has the potential to disrupt biofilms and kill planktonic bacteria as well as other microorganisms while exhibiting low cytotoxic potential [14]. Commonly used cleansing solutions range from potable tap water and sterile normal saline to solutions containing surfactants with or without antimicrobials [14, 27]. Agents such as sterile normal saline are not considered to be effective in removing debris and disrupting biofilms [26, 28], therefore, the use of wound cleansing solutions containing surfactants, e.g., undecylenamidopropyl betaine or phenoxyethanol, gained popularity. Due to their amphiphilic structures, surfactants are able to lower the surface tension between the wound bed and the cleansing liquid. The resulting close contact between the liquid and the wound bed facilitates the separation of nonviable tissue and microbial particles from the viable wound bed. In solution, surfactants additionally capture wound debris in micellar structures [27].

### Antimicrobial active ingredients

Antimicrobials include antiseptics and antibiotics, both having the capability to inhibit microbial growth or to kill microorganisms. While antiseptics are nonselective agents showing a wide antimicrobial spectrum, including bacteria, fungi, and viruses, antibiotics have a narrower spectrum of activity. Antibiotics usually target specific sites within bacterial cells and are therefore relatively nontoxic, since they have no influence on human cells. The adverse consequence is their increased susceptibility to a loss of efficacy caused by bacterial resistance. Antiseptics often have multiple sites of antimicrobial action on target cells; thus, the development of bacterial resistance to antiseptics is uncommon [15, 29]. However, it must not be neglected that antiseptic agents often have a toxic effect to human cells, e.g., on fibroblasts and keratinocytes [30].

### Antiseptics

Antiseptic approaches range from physical treatments to application of synthetic as well as natural substances. Physical treatment methods reduce bacterial burden by directly killing bacteria, including cold plasma and low-level laser therapy [31–35], while synthetic and natural substances decrease the bacterial load by either directly killing bacteria or by inhibiting further bacterial growth.

Elemental silver is relatively inert and has no antimicrobial effect. However, an antimicrobial activity develops after highly reactive positively charged silver ions are released, disrupting bacterial cell walls and inhibiting bacterial enzymes. Since they are also binding DNA, they additionally interfere with cell division and replication [36]. Due to a rapid inactivation of silver ions in the extracellular wound fluid, sustained delivery formulations are required [37].

Iodophors include polyvinylpyrrolidone iodine (PVP-I) and the water-soluble modified starch polymer cadexomer iodine. Free iodine as bactericidal component is gradually liberated from the polymers. Compared to aqueous or alcoholic iodine solutions, these carrier systems lead to lower iodine absorption, reduced cytotoxicity and sensitization, and thus to better toleration [38]. Nevertheless, free iodine still exhibits a relatively high cytotoxicity [30] and the additional risk of thyroid dysfunction after systemic absorption renders the use of iodophores controversial.

The antimicrobial effect of octenidine dihydrochloride (ODC) is based on the interaction of the cationic ODC with the negative charges of cell wall and cell membrane components, resulting in destabilization of microorganism membranes [39]. The comparison of its efficacy and cytotoxicity with other disinfectants like PVP-I showed that ODC exhibits a low cytotoxicity and a high microbicidal effect [40].

Polyhexamethylene biguanide (PHMB), also known as polihexanide, is a strong base and interacts with acidic phospholipids in the membrane of microorganisms, resulting in disruption of the membrane and death of the organisms. Since PHMB is further transferred to the cytoplasm, it additionally disrupts the bacterial metabolism [41]. The advantages of PHMB include a broad antimicrobial spectrum with low toxicity and high tissue compatibility [42, 43].

Acetic acid (AA) is a physiologically active substance that kills bacteria in its nondissociated form [44]. Bjarnsholt et al. showed that AA in physiologically tolerable concentrations is capable to completely eradicate bacteria in mature biofilms in vitro [44].

Hypochlorous acid (HOCl) represents another naturally occurring acid that is physiologically produced by neutrophils to destroy pathogens. HOCL irreversibly binds to sulfur- and heme-containing membrane enzymes as well as to structural proteins, resulting in respiratory loss in bacterial cell membrane leading to cell death [45].

A contemporary antimicrobial approach is the application of naturally occurring enzyme systems that produce antimicrobial products. One example is the oxidase-peroxidase system comprised of glucose oxidase and lactoperoxidase stabilized by the aromatic oil guaiacol (GLG-system) that produces free radicals via the release of hydrogen peroxide, thiocyanate, and hypoiodite. These free radicals mediate cell damage in order to kill microorganisms by oxidizing membranes and enzymes, DNA damage and mutations, and the inhibition of membrane transport [46, 47].

Honey and essential oils are prominent examples for naturally derived substances in wound care. Honey, a viscous carbohydrate-rich syrup, enhances wound healing due to a number of factors such as providing a moist environment and acting as a mechanical barrier for microbes while simultaneously showing analgesic and antimicrobial effects [48]. Essential oils like lavender oil, tea tree oil, and chamomile oil are defined as volatile mixtures of organic and phytochemical components [49]. Observed antimicrobial, antioxidant, and anti-inflammatory properties are explained by the effect of volatile secondary plant metabolites particularly including mono- and sesquiterpenes. Those constituents probably provoke a loss of cellular membrane integrity of bacterial cells which is further related to the observed antimicrobial effects against a wide range of wound pathogens [50]. However, notable disadvantages remain. Honey and essential oils as well as natural extracts consist of a mixture of components, whose composition highly depends on various outer factors such as the plant origin, the geographical location, season variations, as well as processing and storage conditions [51, 52], while many constituents remain unknown [53]. This leads to difficulties in guaranteeing reproducible composition, quality, and quantity of medical products [54]. Additionally, components of such extracts might provoke irritant, allergic, and cytotoxic adverse effects [55–57].

### Antibiotics

Antibiotic therapy of infected wounds includes topical as well as systemic administration. Antibiotics available for topical therapy include aminoglycosides, sulfonamides, and polypeptide antibiotics, as well as metronidazole, fusidic acid, mupirocin, and retapamulin. Aminoglycosides, e.g., neomycin and gentamicin, are effective against most gram-negative and some gram-positive bacteria species like *Staphylococcus aureus*; however, neomycin shows no antibacterial activity against *Pseudomonas aeruginosa* [58]. Like fusidic acid, mupirocin, and retapamulin, aminoglycosides develop their antibacterial effect by inhibiting ribosomal protein synthesis [58]. Fusidic acid penetrates not only intact and damaged skin but also slough and cellular debris and is active against *Staphylococcus aureus* and some other pathogens [29]. Mupirocin as well shows high activity against *Staphylococcus aureus*, including some resistant strains, while being ineffective against most gram-negative bacteria [59]. Retapamulin is bacteriostatic against *Staphylococcus aureus*. By inhibiting the synthesis of folic acid, sulfonamides, e.g., mafenide acetate, silver sulfadiazine, and sulfacetamide sodium, develop a bacteriostatic effect against gram-positive as well as gram-negative bacteria [58]. Polypeptide antibiotics, e.g., polymyxin B, bacitracin, and tyrothricin, disrupt the bacterial cell membrane. While polymyxin b is active against gram-negative bacteria including *Pseudomonas* species, bacitracin can only be used against gram-positive pathogens. The bactericidal activity of tyrothricin includes a broad spectrum of gram-positive bacteria. It has additionally been shown that the polypeptide antibiotic is effective against methicillin-resistant *Staphylococcus aureus* with a reduced susceptibility to mupirocin [60]. Metronidazole causes DNA damage in pathogens and acts against anaerobic bacteria as well as some protozoa [58].

A different perspective is posed by antimicrobial peptides (AMP), host-defence-molecules of multicellular organisms, which aim to control microbial proliferation. Due to the prevalence of basic residues, the majority of AMP is cationic in character and presents an amphipathic structure in membrane-like environments [61]. The mechanism of antimicrobial action is mainly based on electrostatic interaction with the anionic phospholipids of the microbial cell membrane [62], which leads to their disintegration and therefore to cell death [63, 64]. Due to this rather unspecific mechanism of action, the appearance of resistance is less likely compared to conventional antibiotics [65].

## Drug delivery strategies for infected wounds

The use of both antiseptics and topical antibiotics is still often controversially discussed [29, 66]. For successful elimination of infective microorganisms, antimicrobials must reach the anatomical site of infection in adequate concentrations. This is often impeded by potential instability, rapid degradation in the wound environment, or by bacterial enzymes, as well as due to poor penetration into biofilms. Additionally, poor blood circulation in the majority of chronic wounds poses a particular challenge for systemic antibiotic therapy. Inadequate dosing accuracy of systemic antibiotics at the site of infection may further increase antimicrobial resistance and possibly expose patients to unnecessary risk of adverse side effects. Aside the development of resistance, administration of topical antibiotics entails further disadvantages, such as delayed hypersensitivity reactions, superinfections, and contact dermatitis. In the case of antiseptics, inadequate dosing accuracy can further increase cytotoxicity [10] as well as substance-specific adverse effects. However, suitable drug delivery systems overcome the addressed limitations. An optimal formulation of antimicrobials enables the efficient delivery of active ingredients to the site of infection, at the same time supporting the physiological wound healing process.

### State-of-the-art delivery systems

#### Semi-solid and liquid formulations

Traditionally, antiseptic and antibiotic agents have been formulated as semi-solids (ointments, creams, and gels) or liquids (solutions, suspensions, and emulsions) for topical application. Compared to liquid dosage forms, semi-solid preparations for the treatment of bacterial infections remain longer on the wound surface [67]. Ointments are more occlusive and show greater spreading when compared to creams, and therefore, they are used for dry lesions; conversely, creams are more likely to be used for moist lesions [29]. Semi-solid preparations are poorly suited for heavily exuding wounds. While creams quickly absorb fluid, changing their rheological properties and causing leach out of the wound bed, wound exudate impedes close contact of ointments. Solutions are mainly used for therapeutic cleansing of wounds, since they only have short residence time on the wound site, which is further reduced by an excessive excretion of wound fluid [67].

#### Wound dressings

Traditional dry dressings include gauzes, cotton wool, and natural or synthetic bandages. Dry dressings can be used as primary or secondary dressings or as part of a combination of different dressings, with individual functions like absorbing exudate or protecting the wound from external influences. With improved understanding of optimized wound care, the focus shifted towards the development of wound dressings establishing of a moist wound healing environment [67]. Modern wound dressings are additionally capable of enhancing epidermal migration, promoting angiogenesis, providing gas exchange and protecting against pathogens [68].

As there are multiple different types of wounds, a wide range of wound dressings has been developed. Based on wound characteristics and patient condition, a suitable wound dressing must be chosen. Modern dressings are made out of both, natural and synthetic materials, in various physical forms such as films, foams, hydrocolloids, hydrogels, or hydrofibers [67]. Film dressings, historically made out of polyurethane, are extremely flexible, adherent, and transparent. While a certain water vapor transmission rate allows small amounts of exudate to escape through the film, higher amounts of liquid cannot be absorbed and leakage may occur [69]. Polyurethane or silicone foam dressings can absorb large amounts of exudate, but require a secondary dressing for adhesion [70].

Hydrocolloid dressings are made out of gel forming agents such as gelatine, pectin or carboxymethylcellulose, which take up wound exudate, often combined with elastomers and adhesives. They are nonadherent and easy to remove, but their suspected cytotoxic potential limits their application [67, 71].

Hydrogel dressings are crosslinked polymers with hydrophilic structure, such as poly(methacrylates) and polyvinylpyrrolidine. They are capable of establishing a moist wound environment due to their high water content (70–90 %) and also promote autolytic debridement. Nevertheless, their use is restricted by low mechanical strength and low exudate absorption properties [67].

Wound dressings can additionally serve as delivery systems. Commercially available antiseptic dressings include iodine or PHMB containing dressings as well as dressings impregnated with silver. Modern dressings used for the delivery of various antibiotics include collagen sponges and highly absorbing cotton wool dressings [67, 72], however, regulatory approval of such products and the respective access on the international market is quite limited and dependent on the individual regulations of each country.

### Advanced delivery systems

Unfortunately, all delivery systems currently available on the market show certain drawbacks. In case of semi-solid and liquid formulations, the short application period and rapid diffusion processes limit the concentration of antimicrobials at the site of action for the therapeutically intended time interval. Wound dressings impregnated with those formulations can be used to increase the application time; nevertheless, they do neither control the release nor increase the biofilm penetration ability of antimicrobials. In order to meet the requirements mentioned above to a greater extent, a large number of advanced delivery systems has been developed in recent years.

#### Particulate carriers

The potential to penetrate into microbial cells and through the EPS of biofilms has led to an increasing interest in nano-sized particles. While nanoparticles (NP) can directly be applied in form of suspensions, their incorporation in secondary formulations (e.g., hydrogels, sponges, and fibers) eases their application. Table 1 provides an overview of recently developed antimicrobial nanoparticles.

**Table 1:** Summary of recently developed antimicrobial nanoparticles for the treatment of infected wounds

Design of NP	Material	Loading of actives	Secondary formulation	Test model Tested bacteria	Ref.
Metal NP	Silver	-	Wound dressing	In vitro: noncell-based, anti-biofilm assay In vivo: rats Tested bacteria: e.g., *S. aureus*,* MRSA*,* P. aeruginosa*,* E. coli*	[87–92]
	Silver	-	Suspension	In vitro: noncell-based In vivo: rats, mice Tested bacteria: e.g., *S. aureus*,* MRSA*,* P. aeruginosa*,* E. coli*	[81, 93]
	Gold	6-Amino-penicillanic acid	Wound dressing	In vitro: noncell-based In vivo: mice Tested bacteria: *S. aureus*,* E. coli*	[94, 95]
	Gold	Ampicillin, LL37	Suspension	In vitro: noncell-based, anti-biofilm assay In vivo: rats, mice Tested bacteria: e.g., *E. coli*,* K. pneumoniae*,* MRSA*	[96–98]
	Palladium	-	Wound dressing	In vitro: noncell-based Tested bacteria: *E. coli*	[99]
	Copper	-	Suspension	In vitro: noncell-based Tested bacteria: e.g.,* S. aureus*,* P. aeruginosa*,* E. coli*	[100]
Metal oxide NP	Zinc oxide	-	Wound dressing	In vitro: noncell-based Tested bacteria: *S. aureus*, MRSA,* E. coli*	[101, 102]
	Titanium dioxide	-	Wound dressing	In vitro: noncell-based Tested bacteria: *S. aurues*,* P. aeruginosa*,* E. coli*,* B.s ubtilis*	[103]
	Cerium oxide	L-arginin (NO release)	Suspension	In vitro: noncell-based Tested bacteria: *S. aureus*,* E. coli*	[104]
	Copper oxide	-	Suspension	In vitro: noncell-based, anti-biofilm assay Tested bacteria: *S. aureus*,* P. mirabilis*	[105]
	Iron oxide	-	Suspension	In vitro: noncell-based In vivo: mice Tested bacteria: MRSA	[106]
Other inorganic NP	Silica	Gentamicin sulfate	Wound dressing	In vitro: noncell-based Tested bacteria: *S. aureus*	[107]
	Silica	Ampicillin, NO-releasing small molecules	Suspension	In vitro: noncell-based, anti-biofilm assay In vivo: mice Tested bacteria: e.g., *S. aureus*,* S. epidermidis*,* E. coli*	[83, 108]
	Selenium	-	Wound dressing	In vitro: noncell-based Tested bacteria: *S. aureus*,* S. epidermidis*,* E. coli*	[109]
Polymeric NP	Chitosan	Erythromycin, cefadroxil, silver Sulfadiazine	Wound dressing	In vitro: noncell-based In vivo: rats Tested bacteria: e.g., *E. coli*,* S. aureus*,* P. aeruginos*,* B. subtilis*	[110–112]
	Chitosan	Mg2+ / (−)epigallocatechin-3-gallate complex	Suspension	In vitro: noncell-based In vivo: rats Tested bacteria: *S. aureus*,* E. coli*	[113]
	PLGA	Gentamicin sulfate	Wound dressing	In vitro: noncell-based, cell-based Tested bacteria: *S. aureus*,* P. aeruginosa*	[114]
	PLGA	NO-releasing small molecules, levofloxacin, LL37	Suspension	In vitro: noncell-based, anti-biofilm assay In vivo: mice Tested bacteria: MRSA, *E. coli*	[85, 86, 115]
	PCL / pluronic F127	Chloramphenicol	Suspension	In vitro: noncell-based In vivo: mice Tested bacteria: MRSA	[116]
	Gelatin	Selenium (Ru-complex-modified)	Suspension	In vitro: noncell-based In vivo: mice Tested bacteria: *MRSA*,* E. coli*,* S. aureus*,* S. epidermidis*,* P. aeruginosa*	[117]
	Polydopamine	Ciprofloxacin	Wound dressing	In vitro: noncell-based In vivo: mice Tested bacteria: *S. aureus*,* E. coli*,* M. luteus*,* P. vulgaris*	[118]
Other organic NP	Fullerene	-	Suspension	In vitro: noncell-based In vivo: rats Tested bacteria: *S. aureus*,* E. coli*	[119]
	Graphitized carbon black	Vancomycin	Wound dressing	In vitro: noncell-based Tested bacteria: *S. aureus*, MRSA	[120]

*PLGA* Poly(lactic-co-glycolic acid), *PCL* polycaprolactone

Metal or metal oxide nanoparticles are typically used due to their intrinsic antimicrobial activity. The most commonly investigated materials are silver, gold, iron, copper, titanium dioxide, zinc oxide, and cerium oxide [73]. The release of reactive metal ions from the particle surface as well as a generation of reactive oxygen species leading to a membrane disruption of the pathogens are discussed as main mechanisms of their antimicrobial activity [74]. An enhanced efficacy of metal NP instead of materials with greater sizes is attributed to the combined effect of surface attachment and internalization of the NP into microbial cells [75]. Thus, besides the properties of the used material, the particle size as well as shape and surface charge appear to be key factors for determining the predominant mechanism of action [75].

Silver NP, as the most investigated metal NP, have been tested against a broad spectrum of wound pathogens. For instance, Kalishwaralal et al. reported an effect against bacterial biofilms. They assumed that silver NP diffuse through water channels into deeper regions of the biofilm and therefore provide an inhibition of bacterial growth while simultaneously inhibiting the production of EPS [76]. It was further proposed that an interruption of bacterial communication pathways occurs which may lead to a reduced biofilm formation [77].

**Fig. 2:**
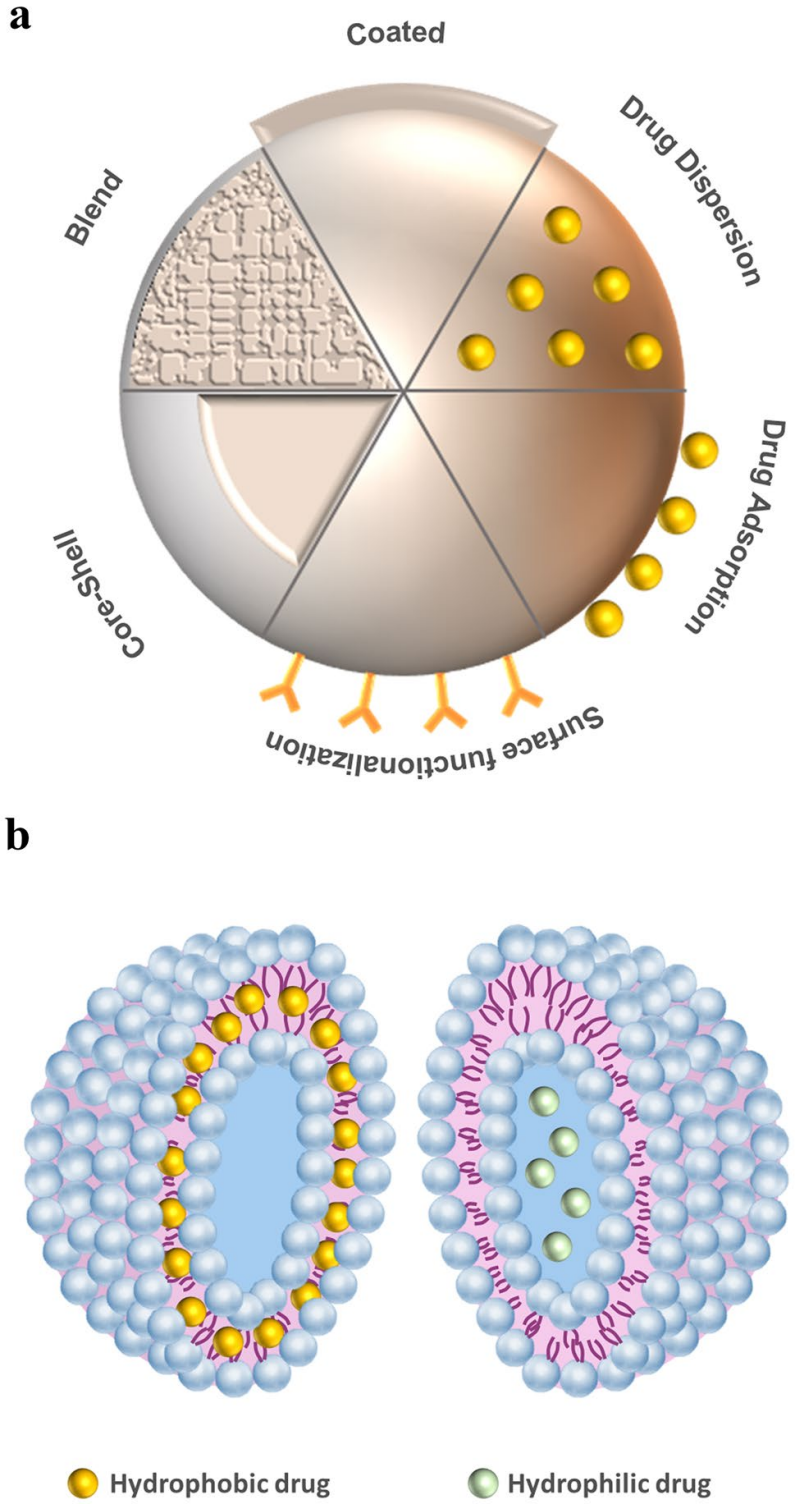
Drug incorporation strategies for particulate and vesicular carriers. **a** Particulate carriers consisting of different materials such as metals or polymers can be modified and loaded with drugs in different ways. **b** Liposomes can incorporate drugs either in the lipophilic membrane or the hydrophilic core

Antimicrobial effects have also been reported for several other metal and metal oxide NP. To enhance the antimicrobial activity or adjust release kinetics, different metal materials are combined in various ways as shown in Fig. 2 [78]. In addition to single metal NP, different metal materials were combined by producing blend NP or NP comprising a core-shell structure with the aim of further enhancing the antimicrobial activity or adjusting release kinetics. Another modification method is doping NP with metal ions to gain an increased potency (Fig. 2) [78].

The inherent properties of NP such as small size and high surface area also provoke disadvantages. Next to the penetration into bacterial cells, NP are also capable of penetrating into human cells, causing cytotoxic effects against human skin cells [79, 80]. However, the cytotoxic activity of metal NP strongly depends on the present concentration and material. To further decrease systemic toxicity, NP were coated with surface stabilizers such as chitosan, leading to an enhanced biocompatibility and a reduced absorption of NP into the blood stream (Fig. 2) [81].

Other inorganic NP serve as drug delivery systems. In the context of the therapy of infected wounds, special attention has been paid to silica NP, due to the fact that their surface chemistry is well known. Their ratio of inert and active functional groups provides a minimal particle aggregation, while still allowing for surface modification and functionalization [82]. Furthermore, NO-delivering silica NP were developed, for which an antimicrobial effect against planktonic and biofilm bacteria could be shown. However, a cytotoxic effect comparable to standard antiseptic therapy was reported [83].

Additionally, organic polymeric NP, which are typically formed from chitosan or poly-(lactic-co-glycolic acid) (PLGA) and loaded with drugs in different ways (Fig. 2), may be promising drug delivery systems. PLGA is characterized through its biocompatibility, biodegradability, and possibilities of controlling the release kinetic [84]. For investigating the influence of the latter on antibiofilm efficacy, Cheow et al. prepared levofloxacin-loaded PCL and PLGA NP exhibiting different antibiotic release profiles and tested their biofilm susceptibility. They concluded that an observed biphasic release profile provides optimal conditions for antimicrobial activity against bacterial biofilms, with an initial burst release to kill bacteria followed by a sustained release over longer time inhibiting biofilm growth and minimalizing exacerbation [85]. PLGA NP have also been loaded with antimicrobial peptides such as LL37. Cherredy et al. produced such NP and reported an accelerated wound closure in comparison to pure LL37 in vivo which was again attributed to a biphasic release profile. Furthermore, antimicrobial activity against *Escherichia coli* in vitro, and no effect to the metabolism and proliferation of keratinocytes was reported [86].

#### Vesicular carriers

A prominent example for vesicular carriers are liposomes, which are formed out of one or more phospholipid double layers and an aqueous core. The amphiphilicity of their membrane offers the opportunity to incorporate both hydrophilic and lipophilic drugs, while providing high biocompatibility and versatility (Fig. 2). The potential of liposomes to fuse with biological membranes or induce their destabilization leads to an enhanced intracellular drug delivery potency [121]. Various active agents have been incorporated into liposomes (Table 2), of which some are discussed in the following.

**Table 2:** Overview of antimicrobial liposomal formulations for the treatment of infected wounds

Material	Loading of actives	Secondary formulation	Test model Tested bacteria	Ref.
Soybean PC, cholesterol, cyanur-PE (lysostaphin conjugated)	Vancomycin	Suspension	In vitro: noncell-based In vivo: mice Tested bacteria: *S.aureus*, MRSA	[127]
DMPC or DPPC or DSPC, cholesterol	Gentamicin	Suspension	In vitro: noncell-based Tested bacteria: *P. aeruginosa*	[124]
DPPC, Cholesterol (+DSPE-PEG-Mal and PE-Rh)	Gentamicin	Chitosan nanofiber mesh	In vitro: noncell-based Tested bacteria: *S. aureus*,* E. coli*,* P. aeruginosa*	[128]
PC	Mupirocin	Chitosan hydrogel	In vitro: noncell-based Tested bacteria: e.g., *S. epidermidis*,* S. aureus*,* B. subtilis*	[129]
PC, oleic acid, cholesterol (pegylated, pyochelin conjugated)	Cefepime, imipenem or ceftazidime	Suspension	In vitro: noncell-based Tested bacteria: *P. aeruginosa*	[130]
DPPC, MSPC, DSPE-PEG-Mal	Ciprofloxacin	Suspension	In vitro: noncell-based, anti-biofilm assay Tested bacteria: *S.aureus*	[131]
PC, cholesterol, tween 80, stearylamine	Bacteriophage cocktail	Suspension	In vivo: mice Tested bacteria: *S. aureus*	[132]
PC	PVP-I	Polyacrylic acid hydrogel	In vitro: noncell-based Clinical study Tested bacteria: *S. aureus*	[122]
Soybean PC	Octenidine dihydrochloride	Suspension	In vitro: noncell-based Tested bacteria: *E.coli*	[39]
DPPC, DSPC, DSPE-PEG-Mal, cholesterol	Trichloroiso-cyanuric acid and cyanuric acid (HClO generating)	Suspension	In vitro: cell-based In vivo: mice Tested bacteria: *S. aureus*	[133]
Soybean PC, polysorbate 20 (neutral) or add. stearylamine (cationic) or soybean PC, sodium deoxycholate (anionic)	Curcumin	Suspension	In vitro: noncell-based Tested bacteria: *S. aureus*,* S. pyogenes*	[134]
Egg lecithin, cholesterol	Epigallocatechin gallate	Suspension	In vitro: noncell-based In vivo: mice Tested bacteria: MRSA	[135]
PC, cholesterol	Propolis	Suspension	In vitro: noncell-based Tested bacteria: e.g., *S. aureus*,* E. coli*,* P. aeruginosa*	[136]

*PC* phosphatidylcholine, *DMPC* 1,2-dimyristoyl-sn-glycero-3-phosphocholine, DPPC dipalmitoylphosphatidylcholine, *DSPC* 1,2-distearoylsn-glycero-3-phosphocholine, *PE* phosphatidylethanolamine, *DSPE-PEG-Mal* 1,2-distearoyl-sn-glycero-3-phosphoethanolamine-N-[maleimide(polyethylene glycol)-2000], *PE-Rh* L-a-phosphatidylethanol-amine-N-(lissamine rhodamine B sulfonyl), *MSPC* 1-stearoyl-2-hydroxyl-sn-glycero-3-phosphocholine, *PVP-I* poly(vinyl pyrrolidone)-iodine

As mentioned before, free iodine exhibits a relatively high cytotoxicity [30] with an additional risk of thyroid dysfunction in case of systemic absorption. To circumvent these problems, Reimer et al. proposed a PVP-I liposome hydrogel for antiseptic treatment of wounds. In cytotoxicity tests, they were able to demonstrate a significantly higher tolerance of PVP-I liposome complexes compared to aqueous PVP-I preparations with identical iodine concentrations. In vitro antimicrobial efficacy tests resulted in a superior antibacterial efficacy of the 3% PVP-I liposome hydrogel compared to a 10% PVP-I ointment. The lower PVP-I content minimizes the potential of systemic absorption, and thus the risk of thyroid dysfunction. The authors conclude that the combination of liposome hydrogel with PVP-I leads to a product that is able to prevent infection, while promoting the wound healing process in form of epithelization due to a moist environment [122].

ODC is another antiseptic for which a liposomal formulation was developed. Not only cleansing solutions but also other ODC agents often contain a surfactant like 2-phenoxyethanol (PE) for the same reasons as discussed before. Since PE has irritating effects, there is need for a new formulation. Szostak et al. introduced a liposomal formulation of ODC, relying on the same positive aspects of liposomes as mentioned above [39].

Next to antiseptic agents, liposomes have also been loaded with a variety of antibiotics. In this context, the possibility of protecting liposome-delivered drugs from enzymatic deactivation was reported. Nacucchio et al. prevented the hydrolysis of the betalactam antibiotic piperacillin by liposomal encapsulation [123]. Mugabe et al. verified this observation, as they reported an enhanced antibacterial effect against gentamicin-resistant *Pseudomonas aeruginosa* from gentamicin-loaded liposomes, proposing that a protection from bacterial enzymes was achieved [124].

However, liposomal formulations struggle with instability issues which can result in drug leakage [84]. The development of polymersomes, another vesicular carrier system, could remedy the instability. By the use of amphiphilic block or graft copolymers with significantly higher molecular weight than lipids, polymersomes form a thicker and therefore much more stable membrane [125]. Polymer nanocapsules have successfully been used to stimulate corneal wound healing and could offer another alternative to liposomal formulations with additional benefits [126].

#### Fibers

**Fig. 3:**
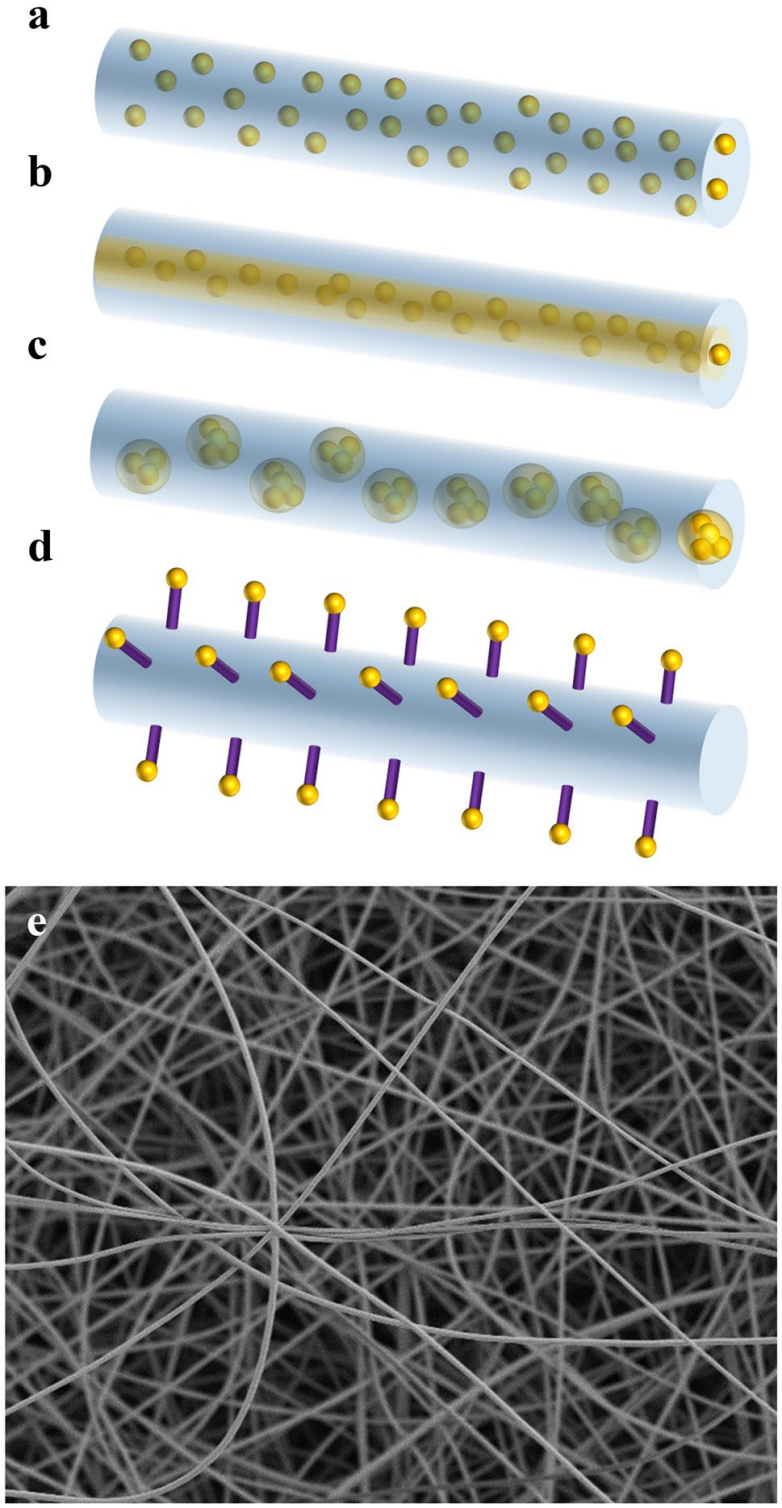
Drug incorporation strategies into fibers. **a** Blend electrospinning, a mixture of drugs, polymers and solvents is directly spun. **b** Co-axial electrospinning, where two solutions are separately spun through a nozzle with two concentric openings. **c** Emulsion electrospinning, where drug solutions are emulsified. **d** Attachment of drugs to the surface by physical or chemical immobilization post-electrospinning. **e** SEM image of electrospun fibers

Fibers with diameters in the nano- to micrometer range have gained increasing interest for wound healing applications. Their fibrous structure closely mimics the human extracellular matrix (Fig. 3), thus favoring cell adhesion, while simultaneously allowing gas exchange, inhibiting microbial infiltration, maintaining a moist environment, and providing high mechanic stability [137].

These fibers are mostly produced by electrospinning, processing a wide range of both natural and synthetic polymers. Of particular interest for the therapy of infected wounds are polymers with inherent antimicrobial activity. In this context, chitosan represents a prominent example. Chitosan is a cationic polysaccharide consisting of a copolymer of glucosamine and N-acetyglucosamine units [138]. Its inherent antimicrobial effect is most likely associated with its cationic nature resulting in interactions with anionic components of bacterial cell membranes, thus inducing an increase of membrane permeability followed by cell leakage and death [139, 140]. Wound dressings consisting of chitosan showed a reduced number of adherent bacteria [141]. Wang et al. further concluded a minimized risk of biofilm formation.

Fibers have been loaded with different antimicrobial agents including classical antiseptics, antibiotics, natural substances, and metal NP (Table 3). Different drug loading methods exist, such as attaching active substances to the surface or embedding them into fibers, as shown in Fig. 3. Attachment of antimicrobial agents to the surface of fibers is achieved by physical or chemical immobilization. This is especially interesting for nanoparticles such as metal NP [142] and liposomes [128]. Monteiro et al. functionalized electrospun chitosan fibers with thiol groups. Thus, a covalent binding of gentamicin-loaded liposomes is enabled. In this way, advantages of electrospun mats as wound dressings are combined with controlled release properties of liposomes [128]. Incorporation of active agents into fibers can be implemented by various methods, such as blend electrospinning, emulsion electrospinning, or co-axial electrospinning. Blend electrospinning is defined by directly spinning a mixture of polymers, actives, and solvents. Spinning of an emulsion in case of emulsion electrospinning can lead to the formation of either a preserved emulsion structure restricted to the inner phase or a core-shell structure through coalescing [137, 143]. Co-axial electrospinning is a direct method to produce fibers with a core-shell structure. In this approach a nozzle with two concentric openings is needed, which inhibits the contact between both solutions until they exit the syringe (Fig. 3) [144].

**Table 3:** Electrospun fibers tested for therapy of infected wounds

Design of Fibers	Material	Loading of actives	Test model Tested bacteria	Ref.
Blend electrospinning	PVP	Ciprofloxacin	In vitro: noncell-based Ex vivo: human skin Tested bacteria: *P. aeruginosa*	[147]
Blend electrospinning	Gelatin, ADA	Gentamicin sulfate and ciprofloxacin	In vitro: noncell-based In vivo: rats Tested bacteria: *P. aeruginosa*,* S. epidermidis*	[148]
Blend electrospinning	Zein	Gentamicin	In vitro: noncell-based Tested bacteria: *S. aureus*,* E. coli*	[149]
Blend electrospinning Coaxial electrospinning	PLA, PLA–collagen or PLA (shell), collagen(core)	Gentamicin	In vitro: noncell-based Tested bacteria: *S. epidermis*,* P. aeruginosa*,* E. coli*	[145]
Blend electrospinning	Gelatin (dopamine crosslinked)	Various polyhydroxy-antibiotics (e.g., daptomycin, vancomycin)	In vitro: noncell-based Tested bacteria: e.g., *S. aureus*, MRSA, *P. aeruginosa*, *K. pneumoniae*, *E. coli*	[150]
Coaxial electrospinning	Pluronic F127 (core), PCL (shell)	Cathelicidin peptide 17BIPHE2 (core)	In vitro: noncell-based, anti-biofilm assay Ex vivo: human skin In vivo: mice Tested bacteria: e.g., MRSA, *A. baumannii*,* P. aeruginosa*	[151]
Blend electrospinning	PU	PHMB	In vitro: noncell-based Tested bacteria: *S. aureus*	[152]
Coaxial electrospinning	PCL (shell) (poly-L-lysine modified)	PVP-I (core)	In vitro: noncell-based Tested bacteria: *S. aureus*,* E. coli*	[153]
Blend electrospinning	PCL	Thymol	In vitro: noncell-based, anti-biofilm assay, cell-based Tested bacteria: *S. aureus*, MSSA, MRSA	[154]
Blend electrospinning	PVP	Curcumin and cerium nitrate	In vitro: noncell-based Tested bacteria: *S. aureus*,* E. coli*	[155]
Surface functionalization	PVA, lysine (Lys)	Lavender oil, ibuprofen	In vitro: noncell-based Tested bacteria: *S. aureus*,* P. aeruginosa*	[156]
Surface functionalization	PCL	Bacteriophage	In vitro: noncell-based Tested bacteria: *P. aeruginosa*	[157]
Blend electrospinning	γ-PGA (ethylene glycol-crosslinked)	Photosensitizer	In vitro: noncell-based In vivo: mice Tested bacteria: *S. aureus*,* E. coli*	[158]

*PVP* polyvinylpyrrolidone, *ADA* alginate dialdehyde, *PLA* polylactic acid, *PCL* polycaprolactone, *PU* polyurethane, *PVA* poly(vinyl alcohol), *γ-PGA* poly(γ-glutamic acid), *PHMB* polyhexamethylene biguanide, *PVP-I* poly(vinyl pyrrolidone)-iodine

The release kinetic of antimicrobials can highly impact their efficacy against bacterial biofilms. To evaluate the influence of drug loading methods on the release kinetic of antibiotics, Torres-Giner et al. encapsulated gentamicin in pure polylactide fibers (PLA), in a blend of PLA and collagen as well as in coaxial fibers consisting of a PLA shell and a collagen core. The desired biphasic sustained release profile could be achieved with core-shell structured fibers. Incorporating the hydrophilic drug gentamicin into pure hydrophobic PLA resulted in a slow release, as a portion of gentamicin was retained inside the hydrophobic structure. The fastest release is observed using a PLA/collagen blend. The water solubility of collagen leads to a porous structure and enables the diffusion of water molecules into the fibers and therefore the release of gentamicin [145]. The observed burst effect of blend electrospun fibers is consistent with another study where tetracycline was incorporated in PVA/chitosan blend fibers [146].

#### Microneedles

**Fig. 4:**
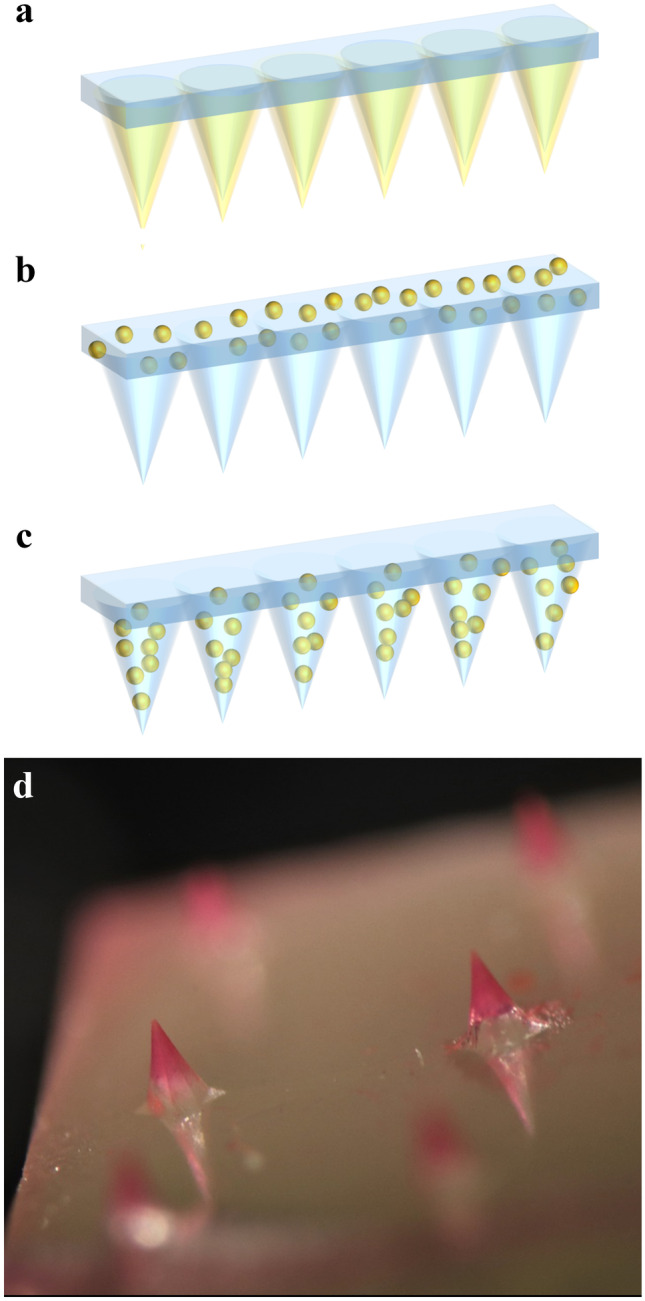
Drug incorporation strategies into microneedles. **a** Coated microneedles. **b** Hydrogel-forming microneedles, the drug diffuses from the patch through the microneedles. **c** Dissolving microneedles, in which the drug is encapsulated. **d** LM image of dye-loaded microneedles

The application of microneedles (MN) is an emerging transdermal drug delivery approach. The microscale needles exhibit a length of 25 to 2000 µm and are arranged on a patch with up to hundreds of needles per centimeter (Fig. 4). It has been shown that MN successfully penetrate the outer layers of skin, while, due to their small size, they do not provoke any pain stimulus and minimize damage to the skin barrier function [159, 160]. The incorporation of active ingredients into microneedles can take place in different ways as presented in Fig. 4. MN made of metal, silicone, or ceramic can be coated with a film containing active ingredients. Hydrogel-forming MN provide another possibility, their gelation enables the diffusion of active ingredients from an attached patch into the skin. A third possibility is posed by dissolving MN consisting of polysaccharides or other polymers in which the drug is encapsulated [161]. While necrotic tissue in infected wounds and the mechanical barrier provided by biofilms often hinder the administration of antimicrobial agents, MN appear to present a promising drug delivery system, since they are able to penetrate those barriers. Recently, several studies examined the effects of MN in combination with various antimicrobial agents on microorganisms and biofilms (Table 4).

**Table 4:** Summary of studies regarding the effects of MN in combination with antimicrobial agents on microorganisms and biofilms

Microneedle design	Fabrication materials	Loading	Test model/tested bacteria	Ref.
Dissolving MNs	PVA, PVP	Silver NPs incorporated into bacteria-responsive microparticles (MP)	In vitro: noncell-based, anti-biofilm assay Ex-vivo: rat skin Tested bacteria: *S. aureus*,* P. aeruginosa*	[165]
Dissolving MNs	Manuka honey	-	In vitro: noncell-based Tested bacteria: MRSA	[166]
Dissolving MNs	PVA, PVP	Carvacrol-PCL-NPs	In vitro: noncell-based Ex-vivo: pig skin Tested bacteria: *S. aureus*,* P. aeruginosa*	[167]
Two-layered dissolving MNs	PVA, PVP covered with PVP, glycerol	Doxycycline loaded PLGA and PCL NPs	In vitro: noncell-based, anti-biofilm assay Ex-vivo: pig skin Tested bacteria: *S. aureus*,* P. aeruginosa*	[163]
Dissolving MNs	PVP	Antimicrobial peptides	In vitro: noncell-based Ex vivo: human skin In vivo: mice Tested bacteria: MRSA, *K. pneumoniae*, *A. baumannii*, *P. aeruginosa*	[164]
Dissolving MNs	Chitosan, Zn2+	-	In vitro: noncell- based, anti-biofilm assay Tested bacteria*: S. aureus*, *E. coli*	[168]
Dissolving MNs with loaded needle tips	PVP	Chloramphenicol bearing, gelatinase-sensitive gelatin NPs	In vitro: anti-biofilm assay Tested bacteria: *V. vulnificus*	[162]
Dissolving MNs	Gantrez® AN-139	Methylene blue	In vitro: noncell-based, anti-biofilm assay Tested bacteria: *S. aureus*,* E. coli*,* C. albicans*	[160]
Dissolving MNs	Hyaluronic acid	Green tea extract	In vitro: noncell-based In vivo: rats Tested bacteria: *E. coli*,* S. typhimurium*,* P. putida*,* B. subtilis*,* S. aureus*	[169]

*PVA* polyvinyl alcohol, *PVP* polyvinylpyrrolidone, *PCL* polycaprolactone, *PLGA* poly(lactic-co-glycolic acid)

Xu et al. fabricated patches with self-dissolvable MN and loaded the needle tips with gelatine NP containing chloramphenicol. After the MN were dissolved in the wound area, the gelatine NP were released and disassembled by the gelatinase produced by the active bacterial community of the biofilm. In comparison to chloramphenicol in free solution, they found that the MN-mediated treatment was more effective in treating *Vibrio vulnificus* biofilms [162]. A similar approach was shown by Permana et al., who investigated the antimicrobial efficacy of chitosan-coated NP loaded with doxycycline and applied with the help of MN [163].

The combination of an electrospun fiber dressing with dissolvable MN arrays and AMP was reported by Su et al. This antimicrobial dressing was not only able to eradicate methicillinresistant *Staphylococcus aureus* (MRSA) biofilms in different wound infection models after daily treatment without applying surgical debridement, but also to completely remove a dual-species biofilm of *Pseudomonas aeruginosa* and MRSA in an ex vivo human skin infection model [164].

Against the background of the state-of-the-art drug delivery systems, the advanced drug delivery systems have some considerable advantages. The penetration into biofilms can be improved by both NP and MN, while liposomes enable the intracellular transport of antimicrobials. Bacterial resistance mechanisms can be overcome by specific vesicular carriers. Additionally, the use of NP and liposomes allows for targeting. Release kinetics can easily be adjusted using polymeric NP or fibers. Fibers further promote physiological wound healing through their structure. However, the field of innovative applications has yet not fully been exploited. Although many promising systems have been developed, the pathophysiological situation of infected wounds as well as requirements for “real life” clinical application are often insufficiently considered, and the studies remain on an academic level lacking transfer potential towards clinical applications. More interdisciplinary research is urgently needed for the development of drug delivery systems that are not only effective in artificial test environments but have the potential to become next-generation wound therapeutics.

## Preclinical testing of novel drugs and formulations for infected wounds

As mentioned above, the simulation of an infected wound scenario represents a particular challenge. Due to ethical and regulatory reasons, wound healing studies with novel drugs or formulations cannot be performed in humans. Therefore, predictive models of human skin wounds, especially in the infected state, are required. Additionally, there is a strong need for appropriate analytical techniques to evaluate the therapeutic effect and potential side effects.

### Models of infected human skin wounds

**Fig. 5:**
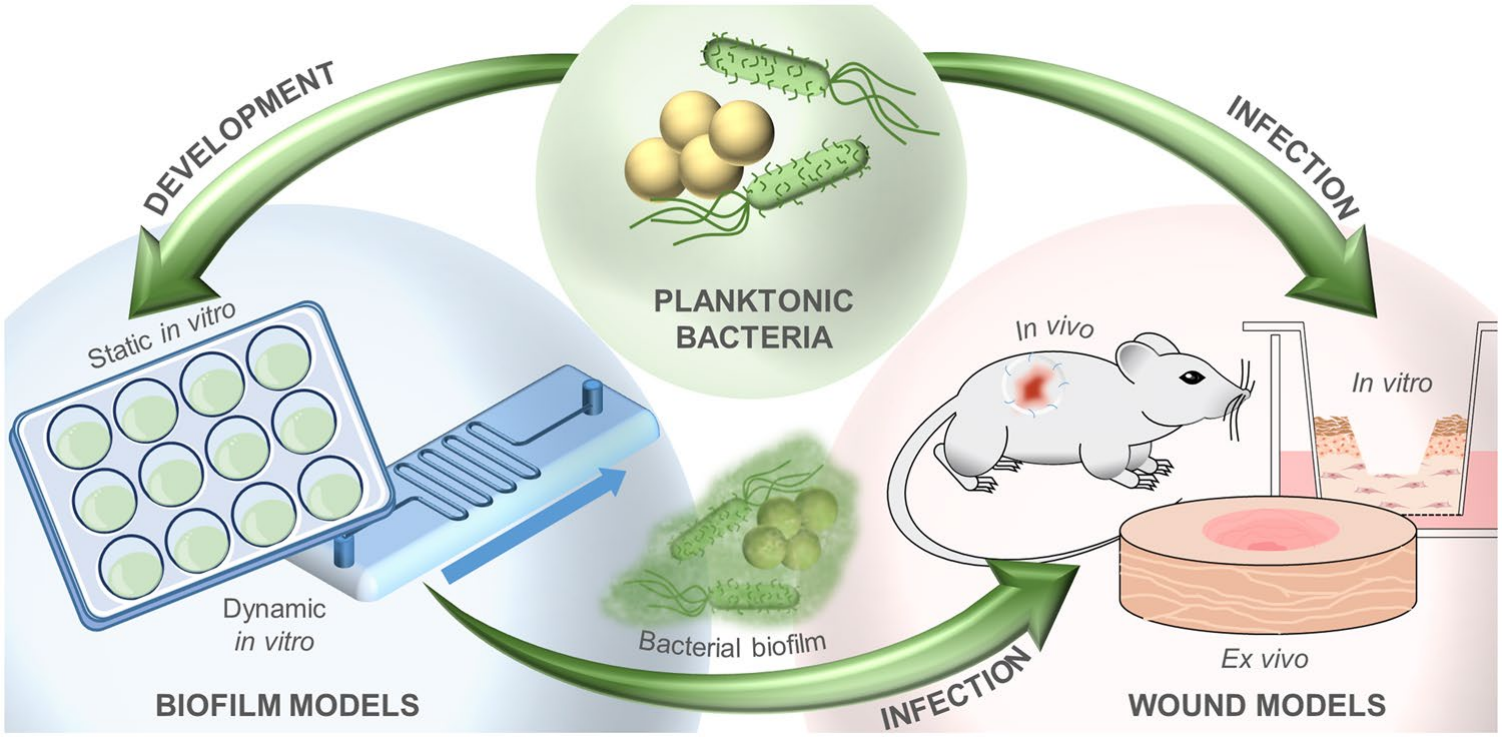
Schematic overview of models of infected human skin wounds: planktonic bacteria can be used to either infect in vivo, in vitro, or ex vivo wound models directly or to develop in vitro static or dynamic biofilm models; precultured bacterial biofilms can also infect wound models

In vivo, ex vivo, and in vitro models have been developed to imitate infected human skin wounds (Fig. 5). Next to the possibility of investigating the wound healing process, chronification of wounds as well as biofilm formation, such models allow for the testing of novel wound healing agents or drug delivery systems. During preclinical testing, animal models are used, including mice, rats, rabbits, and pigs [170]. Even though in vivo models represent a complete organism’s reactions in terms of wound healing, infection, and therapeutic success of applied medications, the translatability of animal data to the situation in the human body is limited. Especially in rodents like mice and rats, which are predominantly used as models, the immune response is significantly different and wound healing is governed by an additional layer of muscles, which is absent in the human body [171]. In this respect, pig models show more similarities to humans [172], but their use is limited due to high costs. Simultaneously, ethical concerns remain [173] and the variability between experiment conditions and different animals results in limited reproducibility and comparability between different animal studies.

Ex vivo infected wound models provide a promising alternative. Porcine or murine tissues are typically used, but models based on human skin also exist [174]. The skin is cultured in an artificial environment (e.g., hydrogel) that offers more controlled experimental conditions [175].

Cell-based in vitro models include two-dimensional cell monolayers and more sophisticated three-dimensional tissue engineered human skin equivalents. Hereby, cell monolayers of keratinocytes offer a very simple method used for basic research purposes, but cannot mimic bacterial invasion and interaction with different cell types or the extracellular matrix that occur in vivo [173, 175]. In contrast, complex three-dimensional models show improved simulation of the complex microenvironment of human infected wounds, but unlike human skin they usually do not contain immune cells as well as blood cells and hair follicles [176, 177].

For simulating an infection, the wound is inoculated with one or more pathogens (Fig. 5), whereby selection of an appropriate concentration for inoculation remains difficult as high doses may induce an increased mortality, while inoculation of too few bacteria may not represent the true status presented in infected wounds and in case of in vivo models pathogen elimination by the hosts immune system may occur [175].

Most models only represent an acute state of infection with planktonic bacteria, while no biofilm formation occurs. This is due to the fact that considering biofilm formation complicates the modeling of infected wounds and requires a prolonged observation period. Still, the main challenge is maintaining an infected wound model over a prolonged period of time in terms of the length of time for chronic wound infections and providing an adequate supply of nutrients and oxygen [175]. To circumvent the need of living cells, static or dynamic in vitro biofilm models can be used to test novel antimicrobial agents (Fig. 5). In vitro biofilm models allow a cheap and easy access to high-throughput experiments. However, a lack of interactions between biofilm and host’s microenvironment exists leading to a questionable physiologic relevance.

### Analytical techniques

The analytical assessment of wound healing can be achieved using a variety of different techniques, with emphasis on optical methods ranging from digital photography to microscopy approaches. Complementary, microbiological assays and DNA sequencing techniques can be used to quantify the bacterial burden.

Light microscopy (LM) is commonly used for histological examination of sample sections. Since it is a low-cost and robust method, the application is widespread [15]. Harrison-Balestra et al. used LM in combination with a modified Congo red staining technique to demonstrate the sequential development of mature biofilm grown by wound-isolated *Pseudomonas aeruginosa*. They could show that the EPS of the developing biofilm was visible after 5 h and exhibited the characteristics of a mature biofilm by 10 h [178]. Since staining only allows for a presumptive identification of species, it is impossible to obtain a definitive identification of microbial species with LM. In addition, its use is limited to microbial cell suspensions and thin tissue sections.

Fluorescence in situ hybridization (FISH) uses fluorescent-labelled complementary DNA, RNA, or modified nucleic acid strands to identify and, if applicable, locate a specific DNA or RNA within a tissue or in a microbial suspension. Imaging is possible with either a fluorescence microscope (FM) or by confocal laser scanning microscopy (CLSM). While the use of FM is limited to microbial cell suspensions and thin tissue sections, CLSM enables the examination of tissue blocks and the reconstruction of 2D or 3D structures. Almeida et al. used a modified FISH method in combination with CLSM to quantify and visualize the different microorganisms in mixed biofilm populations and were able to identify different microbial layers within the biofilm [179]. Further, FISH and CLSM have successfully been used to evaluate the effect of topical ointments on biofilms formed by *Pseudomonas aeruginosa* and *Staphylococcus aureus* [180]. Of course, FM and CLSM can be used with other fluorescent markers [181]; however, the use is limited to the observation of fluorescent structures, while nonfluorescent structures will be missed [15].

Scanning electron microscopy (SEM) enables the identification of biofilm by imaging the surface layers and providing insight into the 3D structure. In vitro biofilms can be investigated regarding size, arrangement, and architecture of cell aggregates and the extracellular matrix [182, 183]. Similar structures in samples from chronic nonhealing wounds in humans can indicate microbial biofilm formation [184]. Since SEM generally requires dehydration of the samples, it is not possible to examine living material; additionally, the dehydration may cause changes in the samples [15].

All these analytical methods have a destructive sample preparation in common, either sectioning and staining or dehydrating. Therefore, the analysis is limited to only one point in time and there is a need for nondestructive methods. Staining has the additional disadvantage that a detailed knowledge of the sample is required so that unknown states can only be discovered by chance. A noninvasive analysis technique widely used in clinical practice is digital photography. The progress of wound healing can be assessed by evaluating the images; however, the photography is limited to superficial observation.

**Fig. 6:**
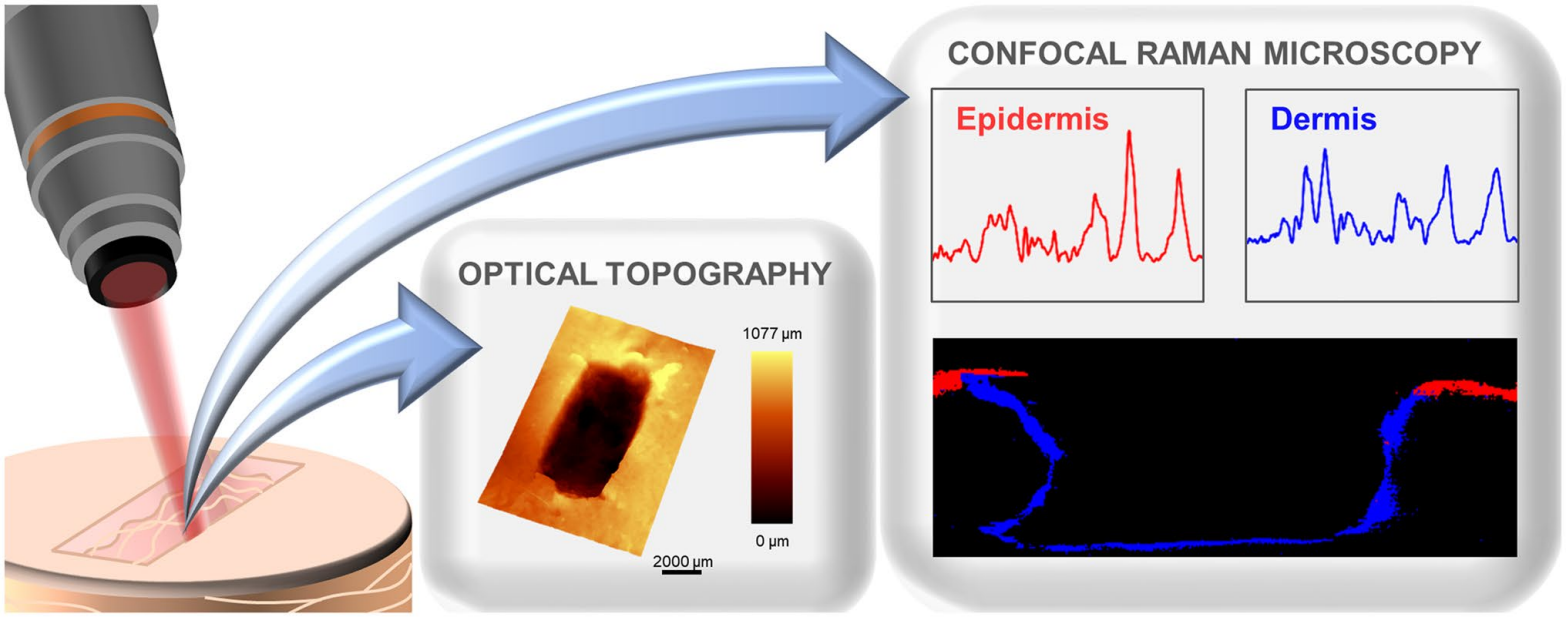
Noninvasive measurement of a wound model, resulting in a three-dimensional visualization of the wound with optical topography and different Raman spectra for the epidermis and dermis, as well as a false-color Raman image of a virtual section of the wound

An emerging technology is optical topography (OT), which enables additional analysis of wound bed volume. Based on the white light reflection, OT enables the nondestructive three-dimensional visualization of the entire defect area within a wound (Fig. 6). Planz et al. reported great potential of this technique for monitoring wound healing by successive scanning of the wound geometry [185]. OT thus represents a technique with which the influence of biofilms on wound healing can be observed noninvasively and over a longer period of time.

Confocal Raman microscopy (CRM) poses another emerging label-free and noninvasive technique, which additionally allows for examining individual cells, biological tissues, and pathophysiological changes within tissues. Raman spectra of biological molecules reveal a chemically selective fingerprint peak pattern, containing information about the sample’s composition and interactions (Fig. 6). Due to these advantages, CRM has already been used for various skin applications, from the analysis of physiological component distribution in skin tissue to the diagnosis of pathological states and the identification of wound healing stages [186, 187]. CRM is a promising technique to monitor the interaction between human skin cells and biofilm at the cellular level and the impact of biofilm on wound healing over time in a noninvasive way.

Further analytical techniques suitable for the noninvasive time-dependent wound analysis are optical coherence tomography (OCT), high-frequency ultrasound (HFUS), in vivo fluorescence laser scanning microscopy (FLSM), magnetic resonance imaging (MRI), multispectral polarized light imaging (MPLI), terahertz imaging (TI), and near-infrared spectroscopy (NIR) [188, 189]. OCT measures the optical pathway from backscattered light to image biological tissue [188, 190, 191]. Kuck et al. assess OCT as insufficiently established to replace histological sections, since it is not possible to reveal the whole complexity of wound healing. However, OCT might offer the possibility of reducing skin biopsies for monitoring purposes [188]. In comparison to HFUS, which uses backscattered ultrasonic waves instead of light, Vogt et al. concluded that OCT shows a better resolution, while HFUS offers the advantage of a less restricted field-of-view [191]. The resolution of FLSM is comparable to histological analysis, though the penetration depends on the laser used and is often limited to the epidermis and upper dermis [189, 192]. MPLI combines the detection of reflection, scattering, and transmission at the same time. MPLI images correlate well with histological analysis and can be generated rapidly over large surfaces, but the resolution does not allow for information of the morphology of individual cells or fine structures [193]. MRI allows for the investigation of the behavior of water molecules in vivo by applying a magnetic field [194, 195]. By capturing pulses of electromagnetic radiation, TPI enables skin areas to be mapped within a few minutes. The thickness as well as the hydration level of the *stratum corneum* can be assessed [196, 197]. IR induces fundamental molecular vibrations, overtone and combination bands of these vibrations can be seen in der NIR region. While IR penetrates only into the uppermost layers of the *stratum corneum*, NIR also penetrates also the dermal skin layer due to shorter wavelengths [198].

## Conclusion and future perspectives

The incidence of infected wounds is increasing, and so is the economic and humanitarian interest in an effective therapy. Biofilm formation especially in chronic wounds complicates the treatment. While there is a wide range of active ingredients, from antiseptics to antibiotics, they are all associated with relevant disadvantages. Aside the challenge of achieving adequate concentrations at the site of infection, bacteria show increasing resistance against many antibiotics. Antiseptics can be used to avoid the development of resistance; however, they are often cytotoxic to human cells. To circumvent these challenges, suitable drug delivery systems are of particular importance. In recent years, the development has moved away from classic pharmaceutical formulations such as semi-solid and liquid preparations in combination with dry wound dressings towards wound dressings that promote wound healing through a moist environment and even more advanced drug delivery systems like particulate carriers, vesicular carriers, fibers, and microneedles. Although these systems are very promising and offer considerable advantages over state-of-the-art drug delivery systems, in most cases, their applicability in clinical practice has not yet been sufficiently considered, resulting in a lack of translation from academic studies to the clinics.

A particular challenge in the development of effective treatments is the preclinical testing to predict the efficacy, absorption, and safety of new active agents or drug delivery systems in an infected wound environment. Besides examination of isolated in vitro biofilm models, different infected wound models ranging from in vivo over ex vivo to in vitro have been developed. However, the imitation of chronic wounds remains a challenge for all models so far. Within the scope of analytics, the noninvasive techniques of confocal Raman microscopy and optical topography represent promising alternatives to established invasive imaging methods.

In addition, the future development of infected wound therapy should not only concentrate on the delivery of drugs adapted to the wound environment, but should also include in situ analysis of wounds with integrated sensors to allow for monitoring of changes in the wound environment.

In summary, there has been a lot of effort in developing effective treatment options for infected wounds. The combination of a better understanding of the wound environment with effective drug delivery systems will enable the future perspective of a targeted therapy, which can slow down the progression of resistance development while at the same time reducing adverse side effects. The area is currently far from being fully developed, but the broad spectrum of novel approaches provides a promising basis for future research.
